# Willingness and ability to pay for unexpected dental expenses by Finnish adults

**DOI:** 10.1186/1472-6831-12-35

**Published:** 2012-08-30

**Authors:** Eeva Widström, Timo Seppälä

**Affiliations:** 1 National Institute for Health and Welfare (THL), P.O. Box 30, Helsinki, 00271, Finland; 2 Institute of Clinical Dentistry, University of Tromso, Tromso, Norway; 3 Government Institute for Economic Research (VATT), Helsinki, Finland

**Keywords:** Utilization of dental services, Willingness to pay (WTP), Ability to pay (ATP), Public sector, Private sector

## Abstract

**Background:**

Since 2002, adults have been able to choose oral health care services in the public sector or in the private sector in Finland. Though various subsidies for care exist in both sectors, the Public Dental Service (PDS) is a cheaper option for the patient but, on the other hand, there are no waiting lists for private care. The aim of this study was to assess middle-aged adults' use of dental services, willingness to pay (WTP) and ability to pay (ATP) for unexpected, urgent dental treatment.

**Methods:**

Postal questionnaires on use of dental services were sent to a random sample of 1500 47-59 year old adults living in three large municipalities in the Helsinki region. The initial response rate was 65.8%. Two hypothetical scenarios were presented: "What would be the highest price you would be prepared to pay to have a lost filling replaced immediately, or, at the latest, the day after losing the filling?" and " How much could you pay for unexpected dental expenses at two weeks notice, if you suddenly needed more comprehensive treatment?" Logistic regression analysis was used to analyse factors related to WTP and ATP.

**Results:**

Most respondents (89.6%) had visited a dentist recently and a majority (76.1%) had used private services. For immediate replacement of a lost filling, almost all respondents (93.2%) were willing to pay the lower price charged in the PDS and 46.2% were willing to pay the private fee. High income and no subjective need for dental treatment were positively associated with the probability of paying a higher price. Most respondents (93.0%) were able to pay a low fee, EUR 50 and almost half (41.6%) at least EUR 300 for unexpected treatment at short notice. High income and male sex were associated with high ATP.

**Conclusion:**

There was a strong and statistically significant relationship between income and WTP and ATP for urgent dental care, indicating that access to publicly provided services improved equity for persons with low income.

## Background

High costs are known to be a barrier for utilization of dental care and many different systems for reimbursement of care costs exist [1-3]. In Finland, dental services are provided both by a public and a private sector. In the public sector, adults’ fees are fixed and subsidized by tax revenues. Private fees have always been unconstrained. However, since 1986, part of private fees has been reimbursed by the National Sickness Insurance, financed by employers, employees and taxation. For half a century, the oral health care system favoured younger people (born in 1956 or later) by providing access to the heavily subsidized Public Dental Service (PDS) and by reimbursing their private costs. The middle aged and elderly were expected to use private dentists or denturists (clinical dental technicians) and pay for the treatments out of pocket. In 2002, a National Dental Care Reform opened the PDS for older adults, too. At the same time subsidization of basic dental care in the private sector by the National Sickness Insurance was extended from young adults to all adults including those born before 1956 [4].

As expected, the reform led to increased demand for dental care by adults, especially in the PDS, where treatment remained cheaper than in the private sector even after the reimbursements from the Sickness Insurance. Long waiting lists for the PDS developed, especially in the capital region where the municipalities had, before the reform, heavily restricted adults’ access to dental care in the public sector, due to the high numbers of private practitioners in the area [5].

A nationally representative clinical epidemiological study in 2000 showed that edentulousness among middle aged adults had decreased considerably during the last 20 years and, treatment needs in this "amalgam generation" had increased. The study also showed that persons with low education and income had greater treatment needs than those with high education and income [6]. There is little information on how the fees charged for dental treatment influence utilization of services and treatment requested by patients.

The aim of this study was to assess use of dental services, and willingness and ability to pay for care and related factors among adults. In particular, we were interested in middle aged adults' willingness to pay (WTP) for urgent dental care and their ability to pay (ATP) for unexpected dental expenses at short notice in two hypothetical situations.

## Methods

Postal questionnaires on the use of dental services were sent to a random sample of 1500 47-59-year-old adults living in the three neighbouring cities of Helsinki, Espoo and Vantaa; they have a total population of one million. A random sample of those born in 1960, 1957, 1954 and 1948 was selected by Statistics Finland. The study was part of the follow-up of the dental care reform in 2002 required by the Ministry of Health from the National R & D Centre for Welfare and Health, which had a legal obligation to collect various data and to monitor developments in the field of social and health care. The inquiry was anonymous and the respondents were under no obligation to complete and return the questionnaires. Approval to conduct the study was given by one of the Directors of the R &D Centre, as was customary when approval by an Ethical Committee was not found necessary. Data collection took place in 2007 and one reminder was sent. The initial response rate was 65.8%. The main questions used in this study were: *"When was your latest visit to a dentist and which treatment sector did you use? What would be the highest price you would be prepared to pay to have a lost filling replaced immediately or, at the latest, the day after the filling was lost?"* and *" How much could you pay for unexpected dental expenses at two weeks' notice, if you suddenly needed more comprehensive care?"* The WTP question was open-ended and the ATP question had structured answer alternatives. In Finland, the PDS is obliged to organize urgent emergency dental treatments the same day. A lost filling does not usually require immediate care, however the condition is irritating for the patient and if left untreated for a longer period endodontic treatment may be needed. In practice, getting an appointment with the PDS could have taken several days or weeks; in the private sector, it could have been possible sooner.

Complete answers to the first and second payment question were received from 704 (46.7%) persons in the original sample. This set of responses was used in the analyses. Background information on the respondents was collected from the questionnaire. Under- or over- representation of certain groups was not observed from the data. However, detailed robustness and representativeness numbers could not be computed due to non-existent data.

Logistic regression analysis was used to model the probability of being willing to pay the same or a higher price than replacement of a lost filling would have cost (after reimbursement) in the private sector (80 €) at the time the study was performed. To assess the factors associated with the ability to pay for more comprehensive treatment, ordered logistic regression analysis was used. This method was chosen because there were five classified responses to the second question: *EUR <50*, *EUR 50-99*, *EUR 100-199*, *EUR 200-299*, and *EUR 300-500*. Gender, basic education (classified in two classes: *comprehensive school* and *matriculation*), professional training (classified in three classes: *vocational qualification (school level)*, *vocational qualification (technical college)*, and *university or equivalent educational level)*, working situation (classified in two classes: *at work*, *off-work*), professional status (classified in six classes: *entrepreneur, upper clerical employee, lower clerical employee, worker, student,* and *other*), annual income (classified in four classes: *EUR <10 000, 10 000-25 000,25 000-50 000* and *>50 000*), year of birth, whether the last dental visit was *in the private*, *in the public sector or elsewhere*, whether the respondent felt a need for dental care, classified as *yes* and *no*, or felt that she/he had benefited from the National Dental Reform classified in *yes a lot, yes a little, no,* and *don’t know*, time since the latest visit to a dentist (classified in two classes: *within the past year, more than one year*) and total costs of dental care in euro in 2006 (or in 2005) were used as explanatory variables. Marginal effects were calculated from the coefficients of the logistic regression and, for the analyses, the level for statistical significance was set at 95%.

## Results

### Use of dental services

A great majority of the respondents (89.6%) claimed to have visited a dentist during the last two years (Table 1). A few persons (2.6%) reported that they had not visited a dentist during the past five years. Most respondents (66.9%) had used private services, 19.6% had used public services and 9.2% had used both sectors.

**Table 1:** Background information on the respondents, the middle-aged adults living in the capital region in Finland, and their use of dental services (n = 704)

**Respondents**	**Male**		**Female**		**Total**	
	**n**	**%**	**n**	**%**	**n**	**%**
**Age**						
47 years	50	18.2	92	21.4	142	20.2
50 years	72	26.3	114	26.5	186	26.4
53 years	75	27.4	123	28.6	198	28.1
59 years	77	28.1	101	23.5	178	25.3
All	274	100	430	100	704	100
**Educational level**						
High	88	32.1	94	21.9	182	25.9
Middle	92	33.6	197	45.8	289	41.1
Low	94	34.3	139	32.3	233	33.1
**Time since the latest visit to a dentist**						
1 year or less	196	71.5	333	77.4	529	75.1
2 years at most	34	12.4	68	15.8	102	14.5
5 years at most	29	10.6	22	5.1	51	7.2
More than 5 years/ or does not remember	14	5.1	4	0.9	18	2.6
**Treatment sector used**						
Private practice	181	66.1	290	67.4	471	66.9
Public Dental Service	56	20.4	82	19.1	138	19.6
Used both sectors	11	4.0	54	12.6	65	9.2
**Mean costs of dental care in 2006 (or 2005)**	€ 262.2		€ 259.7		€ 258.0	
**Work situation**						
Active in working life	224	81.8	361	84.0	585	83.1
Not working	50	18.2	69	16.0	119	16.9
**In need of dental care (own opinion)**						
Yes	132	48.2	204	47.4	336	47.7
No	117	42.7	198	46.0	315	44.7
Does not know	25	9.1	24	5.6	49	7.0

Half of the respondents felt that they were in need of dental treatment (Table1). Only 3.1% were edentulous. The mean cost the respondents claimed to have paid for dental care the year before the study was conducted was EUR 297.4 for those with higher education and, for those with medium or low education, EUR 241.0. However, the difference was not statistically significant. Half (50.2%) of those with higher education, 43.6% of those with medium and 54.4% of those with low education felt that they had benefited financially from the dental care reform. Most respondents were active in working life (Table1).

### Willingness to pay for emergency treatment

In the emergency situation depicted in our study, almost half of the respondents (47.0%) were willing to pay the PDS reference fee (EUR 45) or more but not the private reference fee (EUR 80). A small proportion of the respondents (6.8%) was willing to pay less than the PDS fee. A fifth of the respondents (22.2%) would have paid the private reference fee and 24.0% would have paid even more (Table 2). The highest amount proposed was EUR 300. Persons belonging to higher income classes were prepared to pay more than those belonging to the lower income classes. In the lower income classes women were willing to pay more than the men (Table 2). In the multivariate analysis (Table 3), a high income class and a feeling of having benefited from the dental care reform were statistically significantly and positively associated with the probability of paying a higher fee for emergency treatment. Having a subjective need for dental treatment and latest visit to the public sector or elsewhere (denturist, dental hygienist, hospital) were significantly but negatively associated with willingness to pay a higher price.

**Table 2:** Distribution of respondents according to willingness-to-pay and ability-to-pay groups

**Males, N = 274**						**Females, N = 430**						
	**Income 0-24999**		**Income 25000-**			**Income 0-24999**		**Income 25000-**			**All, N = 704**	
**Willingness-to-pay class, €**	**Between WTP group, %**	**Within WTP group, %**	**Between WTP group, %**	**Within WTP group, %**	**Total, N (row)**	**Between WTP group, %**	**Within WTP group, %**	**Between WTP group, %**	**Within WTP group, %**	**Total, N (row)**	**Total, all (row)**	**Between WTP group, %**
1-44	23.53%	58.86%	5.34%	41.14%	**27**	10.34%	86.00%	1.17%	14.00%	**21**	**48**	**6.82%**
45	10.29%	41.91%	4.85%	58.09%	**17**	13.79%	68.80%	4.30%	31.20%	**35**	**52**	**7.39%**
46-79	38.24%	25.52%	36.89%	74.48%	**102**	48.28%	47.37%	36.33%	52.63%	**177**	**279**	**39.63%**
80	14.71%	18.27%	21.36%	81.73%	**54**	17.82%	30.35%	27.73%	69.65%	**102**	**156**	**22.16%**
81-	13.24%	12.22%	31.55%	87.78%	**74**	9.77%	18.11%	30.47%	81.89%	**95**	**169**	**24.01%**
**Total, %**	**100%**		**100%**			**100%**		**100%**				
**Total, N**					**274**					**430**	**704**	
**Ability-to-pay class, €**	**Between ATP group, %**	**Within ATP group, %**	**Between ATP group, %**	**Within ATP group, %**	**Total, N (row)**	**Between ATP group, %**	**Within ATP group, %**	**Between ATP group, %**	**Within ATP group, %**	**Total, N (row)**	**Total, all (row)**	**Between ATP group, %**
<50	16.18%	73.07%	1.94%	26.93%	**15**	14.37%	73.35%	3.52%	26.65%	**34**	**49**	**6.96%**
50-99	20.59%	46.58%	7.77%	53.42%	**30**	27.59%	64.21%	10.55%	35.79%	**75**	**105**	**14.91%**
100-199	27.94%	35.67%	16.50%	64.33%	**53**	26.44%	42.21%	24.61%	57.79%	**109**	**162**	**23.01%**
200-299	16.18%	26.73%	14.56%	73.27%	**41**	12.07%	39.02%	12.89%	60.98%	**54**	**95**	**13.49%**
300-500	19.12%	9.54%	59.22%	90.46%	**135**	19.54%	21.50%	48.44%	78.50%	**158**	**293**	**41.62%**
**Total, %**	**100%**		**100%**			**100%**		**100%**				
**Total, N**					**274**					**430**	**704**	

**Table 3:** Logistic regression analysis on factors explaining WTP (willingness to pay) the private fee (EUR 80 or more) in a hypothetical situation which required immediate treatment of a lost filling; middle aged adults living in the capital area in Finland

	***Reference level***	***Coef.***	***Std.Err.***	***z***	***P > |z|***	***95% C.I. lower***	***95% C.I. upper***	***Marg.eff (%)***
***Explanatory variable, class***								
Gender, male	Female	-0.069	0.202	-0.340	0.732	-0.465	0.327	4.960
Basic education, Comprehensive school	Matriculation	-0.303	0.248	-1.220	0.222	-0.790	0.183	-7.429
Professional training, Vocational qualification, school level	University or corresp. school level	-0.265	0.365	-0.730	0.468	-0.980	0.450	-6.460
Professional training, Vocational qualification, technical college	University or corresp. school level	-0.275	0.244	-1.130	0.258	-0.753	0.202	-6.745
Working situation, at work	Off work	0.116	0.306	0.380	0.706	-0.485	0.716	2.832
Professional status, entrepreneur	Upper clerical employee	0.502	0.351	1.430	0.152	-0.185	1.189	12.488
Professional status, lower clerical employee	Upper clerical employee	-0.141	0.263	-0.530	0.593	-0.657	0.375	-3.448
Professional status, worker	Upper clerical employee	-0.246	0.315	-0.780	0.436	-0.863	0.372	-5.990
Professional status, other	Upper clerical employee	1.400	0.642	2.180	0.029	0.141	2.659	32.413
Professional status, student	Upper clerical employee	0.748	1.148	0.650	0.514	-1.501	2.998	27.013
Yearly income, 10 k-25 k	<10 k	0.548	0.493	1.110	0.266	-0.418	1.513	13.547
Yearly income, 25 k-50 k	<10 k	1.254	0.514	2.440	0.015	0.247	2.261	30.027
Yearly income, >50 k	<10 k	1.893	0.578	3.280	0.001	0.761	3.025	42.957
Previous visit, public	Private	-1.557	0.281	-5.540	0.000	-2.108	-1.007	4.921
Previous visit, elsewhere	Private	-0.995	0.190	-5.230	0.000	-1.368	-0.623	4.395
Current need, yes	No	-1.158	0.654	-1.770	0.076	-2.439	0.123	10.645
Current need, don't know	No	-1.295	0.375	-3.460	0.001	-2.030	-0.561	6.050
Benefit from reform, yes lot	No benefit	0.073	0.315	0.230	0.817	-0.544	0.689	7.789
Benefit from reform, yes little	No benefit	0.461	0.210	2.200	0.028	0.050	0.871	5.159
Benefit from reform, don't know	No benefit	0.710	0.402	1.770	0.077	-0.078	1.497	17.547
Birth year, 1948	1960	-0.110	0.289	-0.380	0.704	-0.677	0.457	7.066
Birth year, 1954	1960	-0.479	0.275	-1.740	0.082	-1.019	0.061	6.472
Birth year, 1957	1960	0.078	0.273	0.280	0.776	-0.458	0.614	6.758
Previous visit, within 24 months	Within last 12 months	-0.121	0.266	-0.460	0.648	-0.643	0.400	-2.969
Previous visit, more than 24 months	Within last 12 months	-0.290	0.372	-0.780	0.436	-1.018	0.439	8.736
Previous visit, more than 60 months	Within last 12 months	-1.024	0.715	-1.430	0.152	-2.425	0.376	12.506
Same dentist, >0 years	0	0.061	0.296	0.210	0.835	-0.519	0.641	7.246
Constant		-0.199	0.616	-0.320	0.747	-1.407	1.009	N/A

For example, respondents earning more than EUR 50,000 per year had a 43.0% greater probability of exceeding the WTP private fee than those earning less than EUR 10,000. Corresponding proportions for those who earned more than EUR 10,000 but less than EUR 25,000 per year and EUR 25,000 but less than EUR 50,000 per year were 13.5% and 30.0%. Based on logistic regression, there was a statistically significant relationship between income and WTP for private treatment for the two highest income classes. Those who thought free choice between treatment sectors offered a small advantage had 19% higher probability of exceeding the WTP for the private fee than those who thought it offered a large advantage.

The older respondents (born in 1948 and 1954) had a statistically significantly lower probability of exceeding the WTP level of the private treatment price than the comparison group of the ‘younger’ 1960-and 1957-born respondents. Time since the latest dental visit and dental care costs during the latest treatment episode did not have a statistically significant effect on WTP.

### Ability to pay

A small number of respondents (n = 49, 7.0%) reported that the maximum amount they would be able to pay for unexpected dental treatment at short notice was less than EUR 50, 14.9% would have been able to pay EUR 50 – 99, 23.0% EUR 100-199 and 13.5% EUR 200-299. The rest, 41.6% of the respondents would have been able to pay EUR 300 - 500 (Table 2). The mean answer in the middle class was lower (EUR 100-199) and the median was one category above the mean, i.e. EUR 200-299.

As can be seen in Table 2, ability to pay was higher in the higher income groups than in the lower ones for both sexes. In the lower income groups, women would have been able to pay more than men. According to the regression analysis (Table 4), a high income class and being male were statistically significantly positively associated with the probability of being willing to pay a higher price. Previous visits to the public sector or elsewhere and subjective need for dental treatment were negatively associated with the ability to pay a higher price. Respondents earning more than EUR 50,000 per year had a 66% units greater probability of belonging to the highest ATP class compared with those earning less than EUR 10,000.

**Table 4:** Ordered logistic regression analysis on factors explaining ability to pay for unexpected dental expenses at short notice; middle aged adults living in the capital area in Finland

***Expl. Variable, ATP (Ability to pay)***	***Ref. level***	***Coef.***	***Std.Err.***	***z***	***P > |z|***	***95% C.I. lower***	***95% C.I. upper***
Gender, male	Female	0.433	0.166	2.620	0.009	0.109	0.758
Basic education, Comprehensive school	Matriculation	-0.203	0.198	-1.030	0.305	-0.591	0.185
Professional training, Vocational qualification,school level	University or corresp. school level	-0.381	0.287	-1.330	0.184	-0.942	0.181
Professional training, Vocational qualification, technical college	University or corresp. school level	-0.254	0.207	-1.230	0.220	-0.659	0.152
Working situation, at work	Off work	0.019	0.240	0.080	0.938	-0.451	0.488
Professional status, entrepreneur	Upper clerical employee	0.349	0.297	1.180	0.239	-0.233	0.932
Professional status, lower clerical employee	Upper clerical employee	-0.271	0.221	-1.220	0.221	-0.703	0.162
Professional status, worker	Upper clerical employee	-0.421	0.254	-1.660	0.097	-0.919	0.076
Professional status, other	Upper clerical employee	0.524	0.472	1.110	0.267	-0.401	1.449
Professional status, student	Upper clerical employee	0.953	0.970	0.980	0.326	-0.949	2.855
Yearly income, 10 k-25 k	<10 k	0.843	0.361	2.330	0.020	0.135	1.552
Yearly income, 25 k-50 k	<10 k	1.717	0.391	4.390	0.000	0.951	2.483
Yearly income, >50 k	<10 k	2.738	0.464	5.900	0.000	1.828	3.648
Previous visit, public	Private	-0.413	0.205	-2.020	0.044	-0.814	-0.011
Previous visit, else	Private	-0.978	0.443	-2.210	0.027	-1.846	-0.110
Current need, yes	No	-0.498	0.161	-3.090	0.002	-0.813	-0.182
Current need, don't know	No	-0.624	0.295	-2.120	0.034	-1.202	-0.047
Benefit from reform, yes lot	No benefit	-0.057	0.246	-0.230	0.816	-0.539	0.424
Benefit from reform, yes little	No benefit	0.041	0.174	0.230	0.815	-0.300	0.382
Benefit from reform, don't know	No benefit	-0.193	0.310	-0.620	0.534	-0.802	0.415
Birth year, 1948	1960	0.060	0.237	0.250	0.801	-0.406	0.525
Birth year, 1954	1960	-0.045	0.221	-0.200	0.838	-0.477	0.387
Birth year, 1957	1960	0.118	0.225	0.530	0.599	-0.323	0.559
Previous visit, within 24 months	Within last 12 months	-0.411	0.212	-1.940	0.052	-0.826	0.004
Previous visit, more than 24 months	Within last 12 months	0.208	0.293	0.710	0.477	-0.366	0.782
Previous visit, more than 60 months	Within last 12 months	-0.154	0.473	-0.330	0.744	-1.082	0.773
Same dentist, >0 years	0 years	0.648	0.216	3.000	0.003	0.225	1.071

## Discussion

In studies of utilization of dental services so called enabling characteristics such as good dental knowledge and willingness and ability to pay are important because patient contributions are required in most oral health care provision systems, including those with various kinds of subsidies. In health economics, WTP and ATP are hypothetical but direct methods to determinate monetary valuations of effects of health care technologies that have been widely used in a broad range of different diseases [7]. WTP has been proven to be a dexterous tool for assessing and revealing either personal or social preferences or both for matters where data is otherwise inaccessible [8]. In our study, the focus was on personal preferences and the objective was hypothetical.^a^ Hence, while a lot of data on use of dental services and price information can also be relatively easily obtained, in our study the hypothetical focus made the data non-existent. The older middle aged (47-59- year olds) were deliberately chosen for this study because they represent the age groups most in need of dental care in Finland today. They are no longer edentulous but usually have several missing teeth and need comprehensive dental care: restorations, periodontal and prosthetic treatments [6]. Due to the high response rate (for a population study) and because the age and gender distribution in our sample did not differ from the population values in the Helsinki metropolitan area, we consider the material satisfactorily representative of the middle aged population living in the area.

The study showed that frequent use of dental services was more common among adults in the capital region than other parts of Finland [6] which can be explained with on average higher education and better earnings in the population as well as better supply of private dental services in comparison with the rest of the country. In comparison with an earlier study in the same region the proportion of frequent users had increased slightly [9]. Most respondents (76.1%) had used private services and about a third had used public services (28.8%). Although dental care even after reimbursements was considerably cheaper in the PDS than in the private sector, the long waiting lists in the PDS have probably not been attractive to persons used to visiting private dentists with no waiting. Also, private care, after the dental care reform of 2002 [4], should have been less expensive than before. Another possible explanation is that people prefer to go to a dentist they already know. In an earlier study, half (52.9%) of the middle aged respondents had visited the same dentist for ten years or more and only a third (29.5%) for five years or less [9]. In our study, 81.2% of the respondents claimed to have visited the same dentist for more than one year. Private dentists also have recall systems and they send appointments and reminders to their patients [10]. The PDS does not recall adults. A smaller proportion of the respondents claimed to have used both sectors, which in the Helsinki area, most likely means that private patients have used the relatively accessible emergency dental services in the PDS [11].

Replacement of a lost or broken filling was one of the most usual treatments provided in the Public Dental Service Emergency Clinic in Helsinki in 2006 [12] and having access to this kind of treatment was important for an overwhelming majority of the participants in this study, of whom 96.9% retained some or all of their own teeth. In the situation depicted in our study, most respondents (93.2%) were willing to pay a fee that would allow the provision of the necessary fillings. Only 6.8% were willing to pay less and they would probably have chosen extraction. This indicates that most middle aged adults in the capital region put high value on retaining their teeth. It was also obvious that women valued their teeth more than men. Not unexpectedly, willingness to pay a higher price was associated with high income and good oral health. WTP has been shown to be associated with income in other studies about dental care such as periodontal treatments, regular check-ups and dental implants [13-15]. From result-standardization viewpoint but also from the behavioural economics viewpoint, more studies on relationships between predicted and actual WTP are needed.

The other scenario in our study was not as clearly defined as the first and could be interpreted in many ways: a lost or broken crown, bridge or denture, a surgical operation or endodontic treatment. A frequent intervention in the 47-53 year age group would have been endodontic treatment. This, on a molar tooth, would have had a reference price of EUR 150 in the PDS and EUR 360 in the private sector (after reimbursement). According to the results in our survey, 22% of the respondents would not have been able to pay for endodontic treatment and would probably have had the tooth extracted. About 30% could have had endodontics in the PDS and about half of the respondents could have had this care even in private practice. Here it was obvious that those with greater earnings would have been able to spend more money on comprehensive dental care than those with lower earnings. An earlier study in the capital region showed that treatments provided in middle aged adults varied depending on the patientsÂ´ income level. Those with high income had crowns, bridges and implants and those with low income had missing teeth in anterior segments and removable dentures [9].

One of the strengths of our analysis was the possibility of studying and at the same time controlling willingness or ability to pay and income levels. While WTP estimates are sometimes criticised for being biased upwards [16], in our model the ATP and income controlled the bias at least to some extent. In addition, the ATP analysis pointed in the same direction with the WTP analysis and hence provided the results with a more solid basis. In addition, while the positive relationship found between income and WTP and ATP was not surprising, it indicated that, while wealthier people tended to have a preference for the private sector, this preference was not matched to the same extent with their willingness to pay. ATP increased with increasing income, but the increase in WTP did not keep pace with the increase in ATP or income.

## Conclusions

There were strong and statistically significant relationships between income and WTP and ATP for unexpected dental treatments showing that those with high income were willing and able to pay more than those with low income. The recently opened access to the PDS should benefit those with lower income and improve quality of dental care for adults.

## Endnotes

^a^When data are available and accessible, WTP analyses offer little advantage since revealed choices are the most convenient way to study people’s preferences.

## Competing interests

The authors declare that they have no competing interests.

## Authors’ contributions

EW designed and carried out the study and wrote the manuscript. TS contributed to the statistical analyses, interpretation and writing of the paper. Both authors have read and approved the manuscript.

## Pre-publication history

The pre-publication history for this paper can be accessed here:

http://www.biomedcentral.com/1472-6831/12/35/prepub
